# Successful endovascular repair of a ruptured isolated iliac artery aneurysm: A case report

**DOI:** 10.1002/ccr3.2385

**Published:** 2019-08-20

**Authors:** Fumiaki Kimura, Ryo Ookubo, Daita Kobayashi, Hideyuki Harada, Toshio Baba

**Affiliations:** ^1^ Department of Cardiovascular Surgery Kushiro Kojinkai Memorial Hospital Kushiro Japan

**Keywords:** circulatory collapse, endovascular aneurysm repair, isolated iliac artery aneurysm, rupture

## Abstract

While isolated iliac artery aneurysm is rare, its rupture can lead to complete circulatory collapse and possibly death. Herein, we report a case of rupture of a large isolated aneurysm of the right common iliac artery that led to circulatory collapse and rapid endovascular repair saved the patient's life.

## 1 INTRODUCTION

It is common for aneurysms in the iliac artery region to accompany abdominal aortic aneurysms. Conversely, isolated iliac artery aneurysms are quite rare and their causes are not well understood.^1^ The occurrence rate of isolated iliac artery aneurysm, unaccompanied by abdominal aortic aneurysm, is said to be between 0.4% and 1.9% of all aortic aneurysms below the abdominal aorta.^2^

Because they occur deep in the pelvic cavity, isolated iliac artery aneurysms have few subjective symptoms of which patients are aware. Early detection is quite difficult, and if rupture occurs, adhesion to and pressure on the pelvic organs can cause patients to present with morphology involving multiple organs, further increasing the possibility of the condition becoming life threatening. The operative mortality rate of ruptured iliac artery aneurysm has been reported to be 30%‐50%.^2^, ^3^

Open surgery of isolated iliac artery aneurysm can be quite difficult based on the location of the aneurysm and its position relative to other organs. For this reason, endovascular repair has recently garnered attention as an alternative therapy due to its noninvasiveness and reports regarding its favorable results.^4^ In the present case, after diagnosing the patient with a large ruptured right common iliac artery aneurysm with circulatory collapse, we rapidly performed endovascular repair, which saved the patient's life. We report this case as it suggests effective methodologies for dealing with this occasionally encountered, severe pathology.

## 2 CASE HISTORY/EXAMINATION

A 64‐year‐old male patient felt lower abdominal pain 3 days prior to the incident but ignored it. At work, he lost consciousness and was brought to a nearby hospital via emergency transport. Physical examination revealed a difference in blood pressure between his right and left arms, and he was transferred to our hospital for suspected acute aortic dissection. Upon arrival at our hospital, he was conscious and his abdominal pain had been controlled with analgesics. His blood pressure was 96/52 mm Hg, and his pulse rate was 84 beats/min. His medical history revealed untreated hypertension.

## 3 INVESTIGATIONS AND TREATMENT

**Figure 1:**
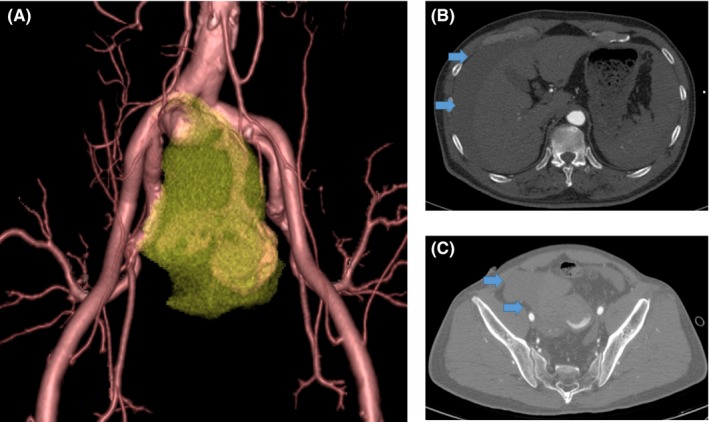
A, Rupture of a 100‐mm‐diameter sacciform isolated iliac artery aneurysm. B, Bloody ascites in the vicinity of the liver (arrow). C, Retroperitoneal hematoma (arrow)

In the emergency room, after placing a central venous line, we performed computed tomography angiography (CTA) because imaging had not been performed at the previous hospital. CTA revealed a 100‐mm‐diameter sacciform isolated right common iliac artery aneurysm. The aneurysm had ruptured toward the retroperitoneal space, and retroperitoneal hematoma and bloody ascites were observed (Figure 1). During examination, the patient's blood pressure gradually declined, ultimately reaching 72/44 mm Hg with a pulse rate of 102 beats/min, indicating shock. His circulation had begun to collapse, and we realized that urgent surgical intervention was necessary. We opted for endovascular repair because the amount of time to prepare for this procedure is comparatively lower than for open surgery. Based on the CTA findings, we decided that embolization of the right internal iliac artery would enable us to properly perform endovascular repair. While maintaining the patient's blood pressure with fluid replacement, he was transported to the operating room as soon as ready. A total of 88 minutes had passed between the patient arriving at our hospital and being transported to the operating room, while 68 minutes had passed between diagnosis confirmation and transport to the operating room.

**Figure 2:**
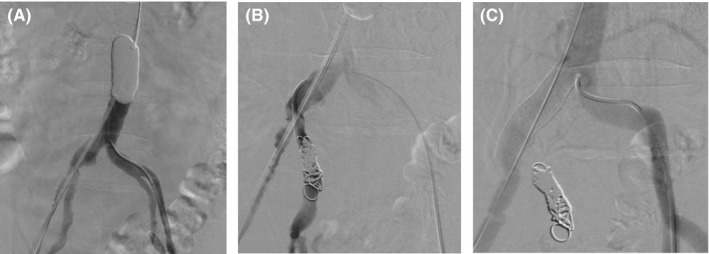
A, Proximal control of the abdominal aorta using an aortic occlusion balloon. B, Coil embolization of the right internal iliac artery. C, Deployment of a stent graft

The operation was performed under local anesthesia and sedation. We made an incision in the right inguinal region, exposed the right common femoral artery, and inserted a 14‐Fr sheath. Then, the left common femoral artery was punctured at the left inguinal region and a 5‐Fr sheath was inserted. Using the 14‐Fr sheath inserted in the right common femoral artery, we inserted an aortic occlusion balloon, inflated it inside the abdominal aorta, and achieved proximal control (Figure 2A). A total of 90 minutes had passed between diagnosis confirmation and proximal control. Consequently, although the patient's blood pressure had declined to 54/36 mm Hg, it recovered to 102/64 mm Hg. Through the 5‐Fr sheath inserted in the left common femoral artery, we inserted a catheter into the right internal iliac artery and performed coil embolization using interlock embolization coils (Boston Scientific; Figure 2B). Next, we removed the balloon inserted in the right common femoral artery and placed a 13‐mm × 5‐cm Viabahn endoprosthesis (WL Gore and Associates) from directly under the abdominal aortic bifurcation to the right common iliac artery and right external iliac artery (Figure 2C). After deployment, the patient's hemodynamics began to stabilize. Prior to the operation, the patient's hemoglobin level had been 10.6 g/dL, but because it declined to 8.7 g/dL, we transfused four units of red blood cells. We closed the incision in the right inguinal region and completed the operation with a total operating time of 69 minutes.

## 4 OUTCOME AND FOLLOW‐UP

**Figure 3:**
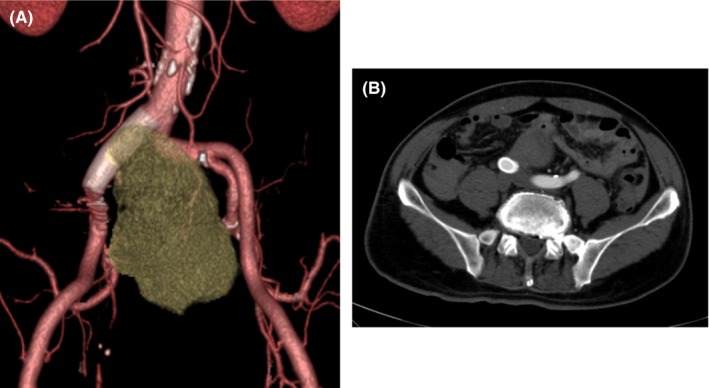
A, After deployment of the stent graft. B, No evidence of endoleak

**Figure 4:**
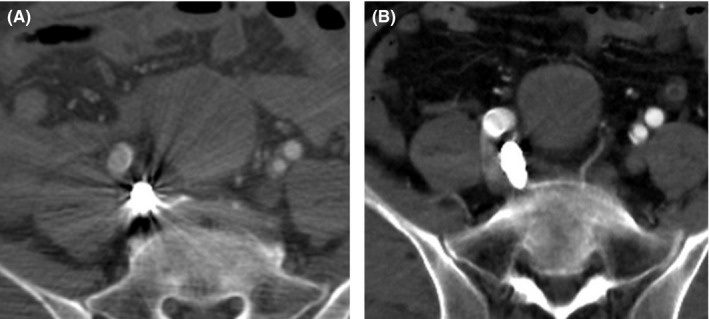
A, CTA at the second day showed 50 mm aneurysm. B, CTA at 1 y after operation showed 40 mm aneurysm

After surgery, the patient was transferred to the intensive care unit and his respiratory and hemodynamics soon stabilized. On the second day, we confirmed via CTA that the aneurysm had thrombosed, and the patient was transferred from the intensive care unit to the general ward. Subsequently, slight subileus occurred, but after fluid replacement and fasting, he soon recovered. On the 24th day, CTA revealed that the aneurysm was excluded and there was no endoleak, and the patient was discharged home (Figure 3). The patient is currently being monitored as an outpatient, and 1 year following the operation, the diameter of the aneurysm has shrinked to 40 mm from 50 mm at the second day (Figure 4).

## 5 DISCUSSION

In this case, a 64‐year‐old man with a 100‐mm‐diameter sacciform isolated right common iliac artery aneurysm suffered aneurysm rupture. Isolated iliac artery aneurysms unaccompanied by abdominal aortic aneurysms are rare,^2^ exhibit few subjectively noticeable symptoms, and are often only detected after rupture occurs. Once they rupture, it is highly likely for circulatory collapse to result, which is invariably life threatening. The operative mortality rate for ruptured iliac artery aneurysm has been reported to be 30%‐50%.^2^, ^3^ Aneurysms with a diameter <3 cm typically grow at a rate of 1.1 mm/y, whereas those with a diameter between 3 and 5 cm have been reported to grow at a rate of 2.6 mm/y. Generally speaking, aneurysms with a diameter >30‐35 mm are suitable for surgical repair.^5^ The average aneurysm diameter in standby cases is 4‐5 cm, while that of ruptured cases is 6 cm; in other words, similar to abdominal aortic aneurysms, the larger the aneurysm, the higher the risk of rupture.^6^ Along these lines, because the diameter of the aneurysm in this case was 100 mm—incredibly large—and because it was in a sacciform shape, we realized that there was a very high risk of rupture.

Treatment methods for isolated iliac artery aneurysm include graft replacement via laparotomy or endovascular repair. In open repair, based on the position of the aneurysm and the presence or absence of internal iliac artery aneurysm, the internal iliac artery may have to be sacrificed or reconstruction. However, it has been reported that aneurysms located deep in the pelvic cavity are very difficult to treat surgically, and the operations to do so have high mortality rates.^7^, ^8^ In the other hand, it has reported that isolated iliac artery aneurysm can be can be safely and effectively treated by open repair or endovascular repair.^9^, ^10^ Moreover, if rupture has occurred, the urgency of the patient's condition, their hemodynamics, and the presence of retroperitoneal hematoma can further complicate the open procedure. Conversely, endovascular repair is very likely to require embolization of the internal iliac artery, bears the risk of intestinal ischemia or buttock claudication, and must be monitored for endoleaks.^11^ However, endovascular repair has the advantage of being far less invasive than the previously described open procedure, has come to be widely practiced, and has achieved results in no way inferior to open procedures.^4^, ^7^, ^8^, ^12^ In particular, in ruptured cases, endovascular repair has the advantage of quickly controlling bleeding and stabilizing circulation. The 2018 Society for Vascular Surgery (SVS) and 2019 European Society for Vascular Surgery guidelines recommend endovascular repair over open surgery if possible.^13^, ^14^ In the present case, the patient's circulation had collapsed and there was a need to quickly control bleeding. Furthermore, imaging allowed us to judge that coil embolization of the right internal iliac artery would make endovascular repair possible, leading us to choose endovascular repair.

In this case, because the patient's diagnosis was confirmed only after arriving at the hospital, he went into shock during examination and his life was in significant danger. The time between arrival at our hospital and entry into the operating room was 88 minutes, the time between diagnosis confirmation and entry into the operating room was 68 minutes, and the time between diagnosis confirmation and proximal control was 90 minutes. According to the 2018 SVS guidelines,^13^ door‐to‐intervention time <90 minutes is strongly recommended, the definition of such time being the time between arrival at the hospital and proximal control. In this case, 111 minutes had elapsed between these two events. The time between CTA diagnosis and admittance into the operating room was 68 minutes; we believe that if that time had been shortened, we could have achieved a door‐to‐intervention time <90 minutes. Because the main reason for the delay was the time taken to prepare the operating room, we believe that keeping surgical equipment necessary for cases of rupture prepared ahead of time can be useful in lessening such delays.

In the present case, because the patient's circulation had already collapsed by the time he was brought into the operating room, we predicted that administration of general anesthesia would have led to further circulatory collapse; therefore, we performed the operation using local anesthesia and sedatives. Lachat et al^15^ previously reported the use of local anesthesia and sedatives in endovascular repair of ruptured cases. Additionally, analysis of the IMPROVE trial indicated an improved 30‐day survival rate with local anesthesia compared with general anesthesia.^16^ We believe that our choice of anesthetic was appropriate given the unstable hemodynamics in this case.

When performing open repair of a ruptured abdominal aortic aneurysm in which circulation is unstable, methods of stabilizing circulation include clamping the descending thoracic aorta via thoracotomy or clamping the abdominal aorta above the celiac artery. However, these procedures take time and tend to be extremely invasive. In recent studies, proximal control using an aortic occlusion balloon has been reported as a useful alternative to these procedures.^17^, ^18^ In the present case, because the aneurysm was isolated and located in the right common iliac artery, we were easily able to use a balloon to achieve proximal control of the infrarenal abdominal aorta. This dramatically improved the patient's hemodynamics, and successful completion of endovascular repair saved the patient's life.

## 6 CONCLUSION

Early detection of isolated iliac artery aneurysm is quite difficult. If detected after rupture, the anatomical characteristics of the condition make surgery difficult, and if circulatory collapse occurs, an exceedingly life‐threatening pathology can result, wherein saving the patient's life may be challenging. However, by rapidly performing endovascular repair, it is possible to stabilize the patient's circulation and significantly improve their chance of survival.

## CONFLICT OF INTEREST

None declared.

## AUTHOR CONTRIBUTIONS

FK: Analyzed data, performed background research, drafted, and revised the manuscript. RO, DK, HH, and TB: Involved in patient management.
